# Neural specialization of print processing in second language learning: A longitudinal ERP study of Chinese children learning English

**DOI:** 10.1016/j.dcn.2026.101691

**Published:** 2026-02-10

**Authors:** Xin Huang, Jai Dellosa Ariza, Shuting Huo, Jason Chor Ming Lo, Catherine McBride, Urs Maurer

**Affiliations:** a School of Psychology, Nanjing Normal University, No.122 Road Ninghai, Nanjing 210093, China; b Department of Psychology, The Chinese University of Hong Kong, Hong Kong, China; c Data Science Institute, University of Wisconsin-Madison, 1205 University Ave, Madison, WI 5370, USA; d Department of Human Development and Family Science, Purdue University, 1202 Mitch Daniels Blvd, West Lafayette, IN 47906, USA; e Brain and Mind Institute, The Chinese University of Hong Kong, Hong Kong, China

**Keywords:** N1 component, Print tuning, EEG, English as a second language, Longitudinal, Word reading skills

## Abstract

The N1 component of event-related potentials (ERP) reflects print tuning and lexicality effects. Previous studies have shown that for a second learned writing system, tuning and lexical effects are associated with age and ability. However, the developmental trajectory of these tuning and lexicality effects, and how language skills influence them, remains unclear. The present study investigated how English reading abilities contribute to longitudinal changes in N1 amplitude and print tuning among Chinese children in Hong Kong. Forty-three children performed a repetition detection task while EEG was recorded to examine three types of tuning: coarse tuning (real word versus false font symbol), fine tuning (real word versus nonword), and lexicality effect (real word versus pseudoword). Children's English word reading accuracy (EWR) was assessed. Results revealed significant coarse tuning and lexicality effects but no significant fine tuning effect. Only coarse tuning showed longitudinal change, with the coarse tuning effect decreasing over time. Furthermore, the N1 coarse tuning effect increased with EWR in the first assessment but decreased with EWR in the second assessment. This modulation was not found for fine tuning or lexicality effects. These findings support the visual perceptual expertise account in the second language (L2) context, demonstrating that the coarse tuning effect, but not fine tuning and lexicality effects, undergoes developmental changes that are modulated by reading skills.

## Highlights

• Coarse tuning effects in L2 English decrease longitudinally in Chinese children.
• English reading ability modulates coarse tuning development, showing opposite patterns between first and second assessments.
• Visual perceptual expertise account applies to L2 contexts, with coarse tuning being uniquely sensitive to development.
• Longitudinal ERP study reveals differential developmental trajectories for three types of neural print tuning in L2 learners.

## Graphical Abstract

**Figure:**
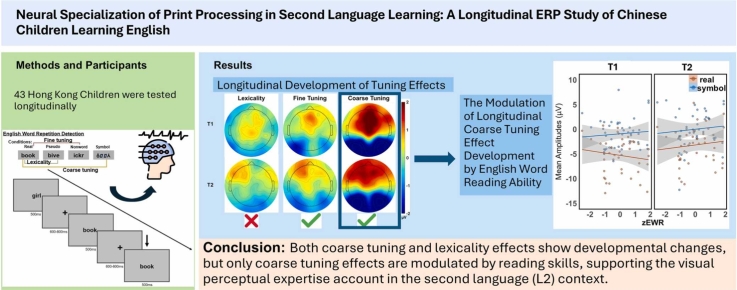

## 1 Introduction

Skilled reading requires rapid and automatic word recognition. This ability relies on neural specialization for print (Schlaggar and McCandliss, 2007), which enables readers to distinguish words from objects and well-formed words from ill-formed words. While the neural mechanisms underlying skilled reading are well-studied in children’s first language (L1), less is known about how these processes develop for a second language (L2). Given the prominence of English in today’s education systems, it is crucial to examine the neural adaptations associated with learning English as an L2, particularly for children whose L1 is rooted in non-alphabetic scripts. The substantial differences between scripts may reflect different developmental processes, raising important questions about the nature of the visual specialization that supports rapid word recognition in English as an L2.

In event-related potential (ERP) studies, the neural specialization for print is believed to be indexed by the N1 component (also called N170 in adults), peaking between 150 and 200 ms, which is typically larger for words than control stimuli such as symbols (Brem et al., 2005, Maurer et al., 2005, Maurer et al., 2008, Maurer et al., 2011, Maurer and McCandliss, 2007, Wong et al., 2005). The left ventral occipito-temporal cortex (vOT) shows distinct activation patterns for print processing, as demonstrated by ERP source analysis (Maurer et al., 2005). This finding aligns with fMRI studies that identified the visual word form area (VWFA) in the left mid-fusiform gyrus, a region that shows specific sensitivity to word forms (Cohen et al., 2000). This neural specialization, often referred to as “coarse tuning for print”, is typically observed for the contrast between words and low-level visual controls (e.g., symbols, pseudofonts). Research indicates developmental differences in the lateralization of coarse tuning for print. While adults display a predominantly left-lateralized coarse tuning effect (Brem et al., 2009, Maurer et al., 2006, Maurer et al., 2010, Xue et al., 2008), children initially exhibit more bilateral activation following early reading training (Eberhard-Moscicka et al., 2015, Zhao et al., 2014), which becomes increasingly left-lateralized with further reading training (Eberhard-Moscicka et al., 2015, Maurer et al., 2006). The N1 component is not only sensitive to coarse print tuning but also to fine tuning effects, referring to the N1 differences between words or pseudowords and nonwords (Araújo et al., 2012, Bentin et al., 1999, Eberhard-Moscicka et al., 2015, Hauk et al., 2006, Zhao et al., 2014), and lexical effects, referring to the neural differences between words and pseudowords (Hauk et al., 2006, Hauk et al., 2006, Maurer et al., 2005, Proverbio et al., 2009).

The N1 print tuning effect is associated with the development of reading ability. Based on the perceptual expertise account (McCandliss et al., 2003), the emergence of perceptual expertise for print or visual word forms is linked to an increased ability to integrate information across the entire stimulus (Rossion et al., 2000). This account is supported by empirical evidence. For instance, a study conducted on German-speaking children with no reading experience (i.e., preschool children) did not show N1 neural specialization, but children with higher letter knowledge showed an atypical N1 print tuning effect even though they could not read, with fast sensitivity to letter strings at right occipito-temporal sites (Maurer et al., 2005). For children in 2nd grade who had mastered basic reading skills, their coarse tuning was stronger for faster readers (Maurer et al., 2006). Similar results were also found in adults. When German-speaking adults performed the same task, they also showed the N1 coarse tuning effect (Eberhard-Moscicka et al., 2016). This suggests that extensive reading experience could facilitate the recognition of familiar visual patterns. Although the N1 print tuning effects are associated with age (e.g., (Eberhard-Moscicka et al., 2016, Huo et al., 2023, Maurer et al., 2006), they showed a dependence on reading skills independent of age (Brem et al., 2009), suggesting that reading ability plays a unique role in N1 print tuning effects.

The development of the coarse tuning effect exhibits an inverted U-shaped pattern, characterized by an enhanced N1 response to print during the initial phase of reading acquisition, followed by a progressive decrease with age (Maurer et al., 2006). Children with developmental dyslexia show a pattern similar to typically developing children, with a robust N1 tuning deficit in the 2nd grade, while in the 5th grade, this deficit has largely disappeared (Maurer et al., 2011). These findings suggest that both typically developing children and those with dyslexia show similar developmental trajectories in their neural responses to print, albeit with different timing.

N1 print specialization typically shows left hemisphere lateralization in adults (Bentin et al., 1999, Maurer et al., 2005, Maurer et al., 2006, Tarkiainen et al., 1999). However, its development follows a distinct pattern. When N1 print tuning first emerges in young children, it shows a bilateral distribution (Eberhard-Moscicka et al., 2016, Zhao et al., 2014). Left lateralization develops gradually with reading instruction and training (Eberhard-Moscicka et al., 2015, Maurer et al., 2006). Right lateralization can occur under specific conditions, such as in pre-reading children with high letter knowledge (Maurer et al., 2005) and in adults learning novel writing systems (Maurer et al., 2010).

The N1 print tuning effects were found in both alphabetic (Eberhard-Moscicka et al., 2016, Mahé et al., 2012, Maurer et al., 2005) and logographic languages (Cao et al., 2011, Zhao et al., 2019). While most studies focused on the N1 print tuning in the first language, only a few studies investigated the effect in the second language (L2) learning (e.g., Huo et al., 2023; Proverbio et al., 2002). Proverbio et al. (2002) found that the occipito-temporal N1 effects were only found for the native language, but not for the languages they acquired later in life, suggesting that this effect is sensitive to both lexical differences and effects of proficiency following foreign language learning. Understanding the N1 print tuning effects in L2 learning is important, as it can provide evidence on how our brain system can cope with different writing systems, especially for children whose native language and second learned language are from alphabetic and logographic systems. Hong Kong Chinese-speaking children are good sample to understand this issue, as they learn to read Chinese and English in parallel, with Chinese having the highest contrast to alphabetic systems. Chinese, as a logographic writing system, differs from English in that it lacks consistent or semi-consistent grapheme-phoneme correspondences. While English establishes clear connections between phonemes and graphemes, Chinese relies on phonetic radicals in most characters, which offer only partial cues to a character's pronunciation (Tan et al., 2001). In addition, as the basic unit of the Chinese writing system, Chinese characters possess a number of strokes that are packed into a square shape, which are visually more complex compared to English letters (Hoosain, 1991). These distinctive features suggest that the neurocognitive processes involved in reading Chinese logographs might vary from those used in reading alphabetic text (Chee et al., 1999, Perfetti and Tan, 1998, Weber-Fox and Neville, 1996).

The N1 component is not only sensitive to the coarse tuning effect, but also to fine tuning and lexicality effect. The fine tuning effect refers to the differences between word forms and illegal letter strings (e.g. consonants, nonwords) (Coch and Meade, 2016, Wang et al., 2023). The N1 fine tuning effect has been found for both adults (Coch and Mitra, 2010) and children (Eberhard-Moscicka et al., 2016, Zhao et al., 2014). The lexicality effect refers to the differences between words and pseudowords (Coch and Meade, 2016, Mahé et al., 2012). Compared to the coarse tuning effect, the N1 fine tuning and lexicality effects were less consistent. Some studies have found a measurable N1 difference between words and pseudowords in German-speaking second graders with an average age of 8.26 years (Maurer et al., 2006). However, more recent research did not identify significant N1 differences between words and pseudowords in 7-year-old German-speaking children (Eberhard-Moscicka et al., 2015, Zhao et al., 2014). Notably, both real words and pseudowords elicited a larger N1 response compared to consonant strings, indicating that fine N1 tuning between real words, pseudowords, and consonant letter strings was present in these 7-year-old children (Zhao et al., 2014).

Most existing research on N1 tuning has focused on L1 processing, with comparatively little attention given to the neural specialization underpinning L2 scripts. Specifically, it remains unclear whether the neural mechanisms involved in L2 script processing reflect a similar pattern to that observed during L1 script processing. One notable study addressing this gap was conducted by Huo et al. (2023), who examined the N1 tuning (coarse tuning, fine tuning, and lexicality effect) to English words in Hong Kong primary school children (grades 1–4), with a focus on the joint contribution of reading ability and age. To assess English reading abilities, Huo et al. (2023) used two tasks: the English Word Reading (EWR) and the English Orthography Knowledge (EOK) task. Their findings revealed that coarse tuning decreased with age but increased with both English task performances, fine-tuning increased with EOK, and the lexicality effect increased with EWR. These results suggest that age and specific reading abilities work differently in terms of driving the N1 neural specialization for a second learned script. Building on these findings, the resent study extends the Huo et al. (2023) study by investigating the developmental trajectories of N1 tuning to L2 script over time. A longitudinal approach will be employed to revisit the same cohort of children at two different time points to provide a more robust understanding of developmental changes by tracking progress over time.

The specific goals of the present study are two-fold:

1) To examine the longitudinal development of coarse tuning effects in second language (L2) processing. Based on findings from first language (L1) studies (Maurer et al., 2011), we hypothesized that the coarse tuning effect would show a longitudinal decrease in L2 learners, as well as the fine tuning effect. Given that the lexicality effects in the N1 component were less robust (Hauk et al., 2006, Hauk et al., 2006, Maurer et al., 2005, Proverbio et al., 2009), no specific hypothesis was formulated regarding their presence or magnitude.
2) To investigate how reading ability influences the development of coarse tuning effects. We hypothesized that EWR at T1 would predict individual variations in coarse tuning both concurrently and longitudinally: better reading ability at T1 would be associated with increased coarse tuning at T1, but a decreased level of coarse tuning at T2. This pattern would be consistent with the inverted U-shaped developmental trajectory observed in a previous study (Maurer et al., 2006).

## 2 Methods

### 2.1 Participants

Data from forty-three native Cantonese-speaking children (16 boys; 1 self-reported as left-handed) were analyzed. Children were assessed longitudinally. During the first assessment, most children were in the second and third grades (2 in Grade 1, 26 in Grade 2, 13 in Grade 3, 2 in Grade 4; mean age 7.91 years old, range 7.05–9.08 years old). The second assessment took place approximately 2.04 years later, at which point most children were in Grades 4 and 5 (5 in Grade 3, 19 in Grade 4, 13 in Grade 5, 6 in Grade 6; mean age 9.95 years old, range 9.06–11.18 years old). From an initial group of 51 children, 5 cases from the first assessment and 4 cases from the second assessment were removed because the average number of artifact-free trials per condition was fewer than 10 (with one participant overlapping in both assessments). In total, 8 cases were removed from the original group. None of the children had been diagnosed with dyslexia. All data were collected in Hong Kong, a highly urbanized city. These 43 children were from an original group of 46 children who were composed of 23 twin pairs. These 43 children were from an original group of 46 children who were composed of 23 twin pairs. The final sample of 43 children included 28 from monozygotic twin pairs and 15 from dizygotic twin pairs. The twin sample was recruited as part of a larger project examining genetic and environmental contributions to bilingual reading development in Chinese and English. While the twin design enables behavioral genetic analyses of reading skills and their neural underpinnings, the current study focuses on developmental changes in neural print sensitivity. To account for data dependence at the family level, all analyses included family as a random intercept, thereby controlling for twin pair membership. Regarding their language education, the students attended schools that primarily used Chinese, while English was included as a subject occupying roughly 20 % of their overall classroom time (Hong Kong Education Bureau, 2002). The background information and assessment results for English reading and language performance are shown in Table 1. The study protocol was approved by The Joint Chinese University of Hong Kong -- New Territories East Cluster Clinical Research Ethics Committee. Consent was obtained from the children's parents. No children were reported to have a history of neurological diseases or psychiatric disorders.

**Table 1:** Demographic and English reading assessment information of the sample.

Variable		*n*	*%*		
**Mother’s education**					
	Elementary school	2	4.65 %		
	Secondary degree or higher diploma	26	60.5 %		
	Bachelor’s Degree	5	11.6 %		
	Graduate Degree	10	23.3 %		
**English use at home**					
	Never	4	9.3 %		
	1–3 times per week	20	46.5 %		
	4–5 times per week	6	14.0 %		
	Almost everyday	11	25.6 %		
**Household income (HKD)**					
	Less than $10,000	4	9.3 %		
	$10,001-$20,000	8	18.6 %		
	$20,001-$30,000	2	4.7 %		
	$40,001-$50,000	4	9.3 %		
	$50,001 or above	25	58.1 %		
		***M***	***SD***	***r***_***AgeT1***_	***r***_***AgeT2***_
**Age in T1**		7.91	0.57		
**Age in T2**		9.95	0.62		
**English word reading (max = 50)**		21.16	15	0.39	0.34
**English rapid digit naming (secs)**		2.12	0.65	0.35	0.32
**English vocabulary knowledge (max=48)**		25.51	11.81	0.34	0.29
**English dictation (max =36)**		8.7	6.8	0.5^***^	0.45^**^

Note: ***. Correlation is significant at the 0.001 level (2-tailed).

**Correlation is significant at the 0.01 level (2-tailed).

Details of English rapid digit naming, English vocabulary knowledge, and English dictation are provided in the supplementary materials.

### 2.2 English word reading ability

The study used an English word-reading assessment previously used with Chinese ESL students (Tong and McBride-Chang, 2010, Zhou and McBride, 2018). Fifty words were selected from English textbooks, including 41 regular words that followed standard grapheme-phoneme correspondence patterns (Earle and Sayeski, 2017). Based on pilot testing, words were arranged by difficulty, with multisyllabic and less frequent words considered more challenging. Participants continued reading aloud until making 5 consecutive errors, receiving one point per correct pronunciation. The assessment demonstrated high reliability with a Cronbach's alpha of 0.91. Reading ability was assessed at T1.

### 2.3 Experimental procedure

**Fig. 1:**
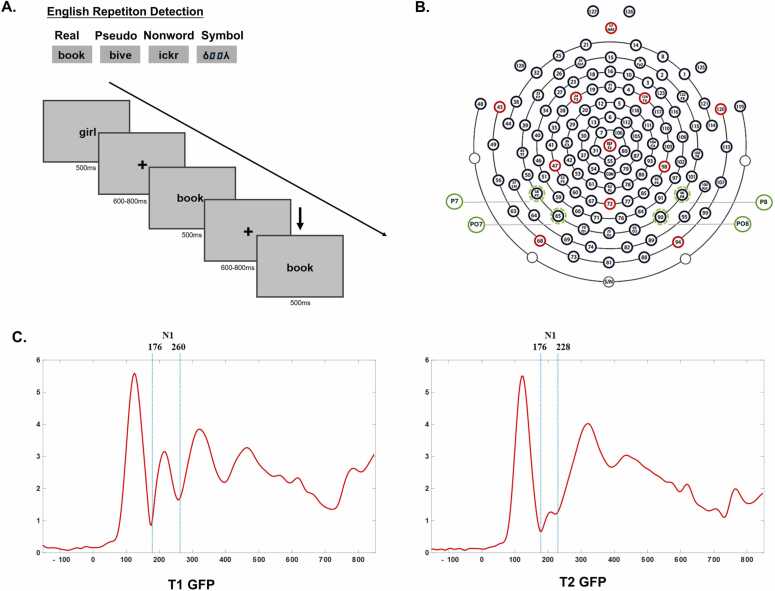
A) Illustration of the repetition detection task with examples of stimuli. B) 128- electrode positioning and regions of interest (green ones), C) Global field power averaged across four conditions for T1 and T2.

The study implemented a one-back repetition detection paradigm (following Maurer et al., 2005, Maurer et al., 2006; Tong et al., 2016), comprising four experimental conditions: real words (high-frequency English words), pseudowords (phonetically and orthographically valid letter combinations without semantic meaning), nonwords (orthographically illegal letter combinations derived from the real word stimuli), and false font symbols (unrecognizable graphical conversions of real word stimuli, following Eberhard-Moscicka et al., 2015) (Fig. 1A). All verbal stimuli consisted of four letters. Each condition contained 58 unique stimuli and 8 repetitions, totaling 66 trials. Trials where stimuli were repeated (i.e., target trials requiring a button press) were excluded from EEG analyses. Only non-repeated trials were included to ensure that the neural responses reflected pure stimulus processing without confounding effects of repetition detection or motor preparation.

The experimental protocol presented stimuli centrally on a computer display with black characters against a silver background. Each trial commenced with a fixation cross (600–800 ms, randomized duration), followed by a 500 ms stimulus presentation. Participants were instructed to indicate stimulus repetitions via button press on an SR Box. Stimuli were presented at 56-point font size, subtending vertical visual angles of 1.32° to 2.28° and horizontal visual angles of 2.08° to 4.29°.

The experimental design comprised 264 trials (66 trials × 4 conditions) organized into eight blocks, with each block subdivided into two counterbalanced sub-blocks of 33 trials (including two repetitions). Inter-block intervals were 30 s. Stimulus presentation and response recording were implemented using E-Prime software.

Testing was conducted individually in a sound-attenuated EEG laboratory. Prior to data collection, a Cantonese-speaking research assistant trained in EEG procedures provided detailed instructions to each participant. Participants received demonstrations of EEG waveform artifacts induced by movement and blinking to facilitate compliance with experimental requirements regarding stillness and ocular control. A practice session was administered to ensure task familiarity before commencing the experimental protocol.

### 2.4 EEG recordings and preprocessing

The EEG recordings were acquired using a 128-channel HydroCel GSN EGI system (EGI net station v5.3, Electrical Geodesics Inc., Eugene, Oregon), utilizing a 500 Hz sampling rate and online Cz electrode reference. Electrode impedances were maintained below 50kΩ throughout recording, and the time delay (12 ms) between the event trigger and the actual stimulus onset was corrected based on a separate timing test.

Signal processing was executed through EEGLAB 2020.0 (Delorme and Makeig,2004), employing an optimized preprocessing sequence, which was adapted from Lo et al. (2019). This pipeline has been examined and compared to several alternative pipelines, including the Harvard Automated Processing Pipeline for Electroencephalography (HAPPE; Gabard-Durnam et al., 2018), Makoto's preprocessing pipeline, and the NetStation officially recommended pipeline. Compared to these alternative approaches, the current pipeline showed marginal gains in signal-to-noise ratio (SNR) and demonstrated validated performance in automated independent component labeling and consistency.

Initial signal conditioning involved band-pass filtering (0.3–30 Hz) and conversion to a standardized 10–10 montage system to address peripheral electrode instability. The data underwent down-sampling to 250 Hz, followed by automated identification of high-variance channels using PREPpipeline (Bigdely-Shamlo et al., 2015). Signal decomposition was accomplished through the infomax ICA algorithm (Bell and Sejnowski, 1995), with subsequent automated removal of ocular artifacts via the ADJUST algorithm (Mognon et al., 2011). The continuous data were then segmented into epochs (-150–850 ms), followed by spherical spline interpolation of excluded channels. Final processing steps included average reference transformation, baseline normalization (-150–0 ms), and condition-wise averaging. This processing pipeline was selected based on superior signal-to-noise characteristics compared to alternative preprocessing approaches. The mean (SD) SNR in T1 was 11.7 (3.7) for P1 and 10.3 (4.7) for N1, while in T2, it was 13.5 (5.6) for P1 and 9.5 (3.7) for N1. The mean (SD) number of trials for the four conditions in T1 were Real: 37.5 (10.1), Pseudo: 36.3 (9.9), Nonwords: 36.9 (10.4), False font symbols: 37.3 (10.3). In the second assessment (T2), the mean number of trials for the four conditions were Real: 40.5 (9.9), Pseudo: 40.5 (10.4), Nonwords: 39.8 (10.4), False font symbols: 40.6 (9.5). A repeated-measures ANOVA with Time and Condition as factors revealed no significant main effects or interactions for the number of trials retained (*F*s < 3.39, *p*s > .07).

Participants for data analysis were chosen based on the following criteria: 1) a Signal-to-Noise Ratio (SNR) above 3 on the expected P1 or N1 event-related potential (ERP) components; 2) a score above 70 % on the repetition detection task; 3) at least 10 trials for each of the four conditions. SNR was calculated using the following procedure: (1) the mean signal across all electrodes was computed at each time point to obtain the Global Field Power; (2) the standard deviation of the global mean field was calculated as the noise estimate; (3) peak amplitude was identified within the component time window (50–150 ms for P1, 150–250 ms for N1) from the global field power; and (4) SNR was computed as peak amplitude divided by the noise estimate (standard deviation). Consequently, 8 participants were excluded due to an insufficient number of trials.

### 2.5 Data Analysis

The selection of regions of interest (ROI) for the N1 component was based on previous literature (e.g., Maurer et al., 2008; Tong et al., 2016; Huo et al., 2023), which indicated that the occipitotemporal regions (PO7, PO8, P7, P8; see Fig. 1B) showed the largest activation. The selection of time window was based on global field power (GFP, see Fig. 1C), which was 176–260 ms in T1, and 176–228 ms in T2.

We analyzed ERP time-locked to the target words using linear mixed effect model (LMMs). When we ran the models, we always began with full models that included the maximum random effects structure. But the slopes were removed if the model failed to converge (indicating over-parametrization). Compared to factorial ANOVAs, LMMs with maximal random effects structure have the best potential to produce generalizable results (Baayen et al., 2008, Barr et al., 2013). N1 amplitude (averaged across the time window at occipitotemporal sites) and lateralization (amplitude difference between left and right sites) were analyzed separately. Each model included the following fixed effects: the three-way interaction between time, EWR, and condition together with embedded two-way and one-way contrasts. The raw EWR scores were transformed into z scores. Regarding the condition effect, the real word condition was set as reference while prespecifying three tuning effects. Trial order (centered) was entered as a fixed effect because previous findings showed a repetition effect with later trials eliciting stronger negativity (Yum et al., 2018). In addition, age (centered) was also included as a fixed effect as age strongly influenced the N1 amplitude (e.g., Huo et al., 2023; Tong et al., 2016).

The random effects were identified through an iterative approach, as recommended by Baayen (2008). A random slope was included only if it significantly enhanced the model's goodness of fit. Family ID was used as a random intercept to address data dependence within twin pairs, while Participant ID and Item were included as random intercepts to account for the crossed design. We specified three contrasts for the factor of condition in the model, including real-symbols, real-pseudoword, and real-nonword contrasts. The complete representation of the complex LMER model for N1 amplitude is provided in the R code below. The model for N1 lateralization was identical to the model for N1 amplitude.

Amplitude/N1 laterization ∼ zAge + zOrder + time*condition*EWR + (1|Subject) + (1|Item) + (1|FamilyID)

For behavioral data, LMER was employed to analyze fixed and random effects on response time in the repetition detection task at the trial level. For accuracy, general linear mixed effect regression (GLMER) with a Bernoulli distribution for residuals was adopted to predict the probability of giving a correct response to each trial. The random effects for the LMER model for accuracy and response time included Item, Participant ID, and Family ID as random intercepts. The model for behavioral data is provided in the R code below.

Reaction time/ACC∼ zAge + zOrder + time * Condition * EWR + (1|Subject) + (1| FamilyID) + (1|Item)

The analysis was conducted with the lmertest package (Kuznetsova et al., 2017) in the R computing environment (R Core Team, 2023). The LMER model was estimated using the restricted maximum likelihood (REML) method. For a priori contrasts (three types of print tuning), the multivariate t (MVT) method (Genz and Bretz, 1999) computed the adjusted p value for each contrast while keeping the overall alpha level at 0.05. Subsequently, the post hoc analyses were motivated by significant contrasts. The p values were adjusted using the Bonferroni approach to 0.025 (0.05/2). Variable Inflation Factors (VIF) for the subject-level variables were 1.00 (time), 1.03 (EWR), and 1.01 (interaction between time and EWR). The values were all smaller than the conservative threshold of 2.5 (Johnston et al., 2018), suggesting there was no issue of multicollinearity.

## 3 Results

### 3.1 Behaviour results

Table 2 presents the means and standard deviations of accuracy rates and reaction times across all conditions. The model incorporating English Word Reading (EWR) demonstrated a significant time effect: the probability of correct responses in the second assessment was significantly higher than in the first assessment (*В* = 1.89, 95 % *CI* [1.54, 2.32], *p* < .001). Furthermore, participants showed significantly higher accuracy in the real word condition compared to the symbol condition (*В* = 0.26, 95 % *CI* [0.13, 0.49], *p* < .001). Analysis revealed a significant interaction between the word-symbol contrast and EWR (*В* = 0.59, 95 % *CI* [0.41, 0.85], *p* = 0.005), suggesting that children with higher word reading ability showed a more pronounced word effect relative to symbols. However, no significant three-way interaction was found among time, condition (all three contrasts), and English word reading (all *p* > .05).

**Table 2:** Descriptive statistics for behavioral performance in the repetition detection task (SD in parentheses).

**Time**		**Condition**	**Mean Accuracy (SD)**	**Mean RT (SD)**
T1		real	0.98 (0.12)	740.84 (418.71)
		pseudoword	0.97 (0.16)	793.07 (326.62)
		nonword	0.98 (0.14)	806.47 (431.03)
		symbol	0.95 (0.21)	859.50 (336.53)
T2		real	0.99 (0.10)	778.82 (254.69)
		pseudoword	0.99 (0.11)	964.00 (354.79)
		nonword	0.99 (0.10)	823.88 (389.75)
		symbol	0.97 (0.16)	838.17 (290.61)

Regarding reaction time, responses to real words were significantly faster than to symbols (*В* = 28.27, 95 % *CI* [13.54, 43.01], *p* < .001). A significant time effect was also observed, with children responding more quickly in the second assessment compared to the first (*В* = −9.40, 95 % *CI* [-12.41, −6.39], *p* < .001). Additionally, there was a significant interaction between time and the real-symbol contrast, with the effect diminishing as children aged (*В* = −15.19, 95 % *CI* [-23.58, −6.79], *p* < .001). The interaction between EWR and the lexicality effect (real vs. pseudowords) was significant, with children having higher EWR ability showing a smaller lexicality effect (*В* = −6.19, 95 % *CI* [-12.23, −0.15], *p* = 0.044). Children's English reading ability significantly improved with age (*В* = 4.87, 95 % *CI* [1.88, 7.86], *p* = 0.001). All other contrasts and their interactions were non-significant (all *p* > .05).

### 3.2 EEG results

**Fig. 2:**
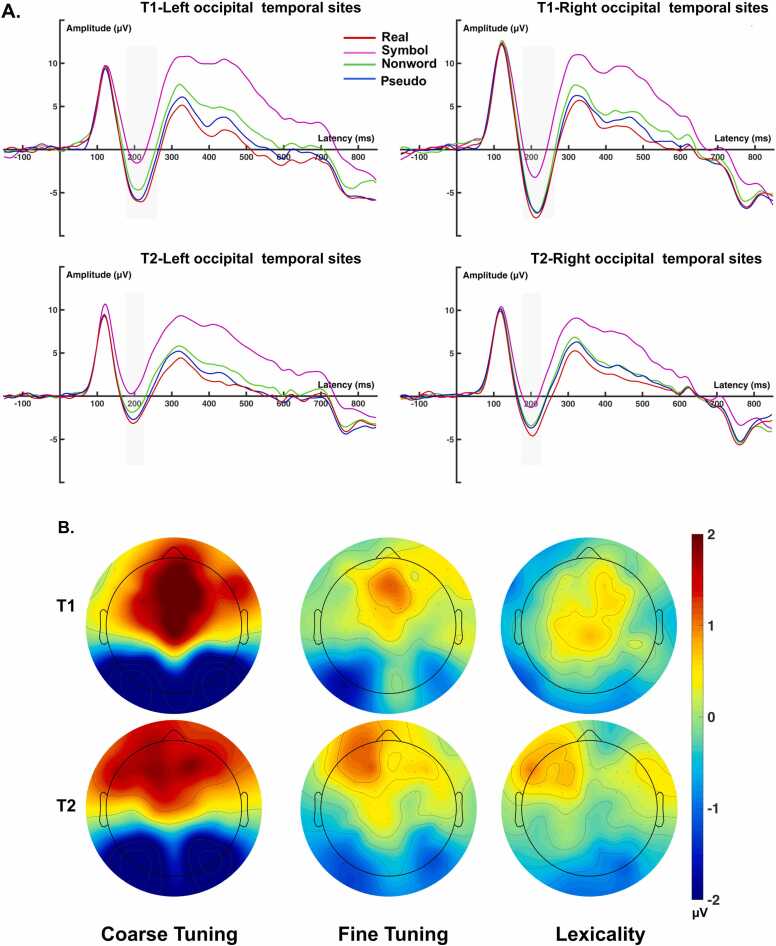
A) ERP waves at four conditions at left and right sites with time windows used for analysis (light gray regions). B) Topographic maps for difference waves for N1 at three tuning effects.

**Fig. 3:**
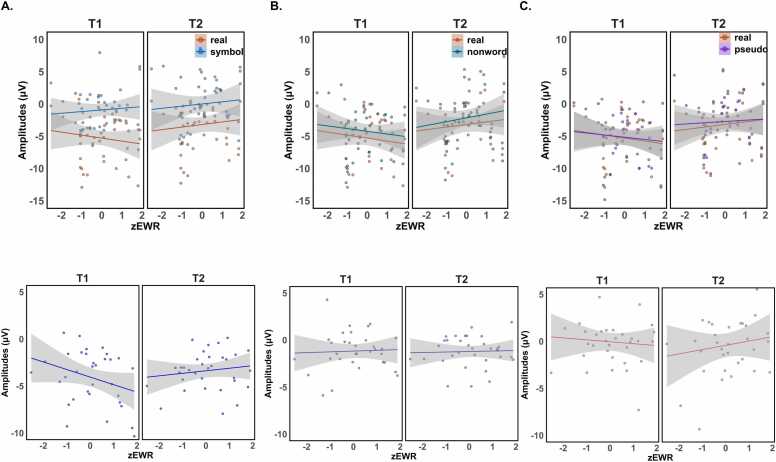
Scatter plots and estimated effects of ability on tuning effects and N1 amplitude in relevant conditions, i.e., simple effects (below. A) English word reading ability and coarse tuning, B) English word reading ability and fine tuning, C) English word reading ability and lexicality effect.

The ERP results revealed a significant coarse tuning effect (see Fig. 2 for waveforms and topography maps; real vs. symbol; *В* = 4.57, 95 % *CI* [3.91 – 5.24], *p* < 0.001) and fine-tuning effect (real vs. nonword; *В* = 1.16, 95 % *CI* [0.50 – 1.83], *p* = 0.001). In addition, the coarse tuning effect decreased from the first assessment to the second assessment (*В* = −1.23, 95 % *CI* [-1.96 – −0.49], *p* = 0.001). Furthermore, the three-way interaction of the coarse tuning effect with time and EWR was also significant (*В* = −0.81, 95 % *CI* [-1.53 – −0.08], *p* = 0.03; see Fig. 3A). Post hoc analyses for each condition at each assessment revealed that as EWR scores increased, N1 amplitudes significantly decreased in response to real words at the first assessment (*В* = 0.70, 95 % *CI* [0.19 – 1.22], *p* = 0.007). However, no significant changes were observed in N1 amplitudes for symbols (*В* = −0.02, 95 % CI [-0.56 – 0.51], *p* = 0.94). The post hoc analyses for each assessment showed that the N1 coarse tuning effect increased with EWR in the first assessment (*В* = 0.61, 95 % *CI* [0.04 – 1.17], *p* = 0.034), but the decreasing trend with EWR in the second assessment was not significant (*В* = −0.16, 95 % *CI* [-0.62 – 0.31], *p* = 0.50), resulting in the significant longitudinal modulation of EWR on N1 coarse tuning.

The two-way or three-way interactions involving the fine-tuning effect were not significant (all *p* > 0.05). The main effect of time was significant (*В* = 2.07, 95 % *CI* [1.81 – 2.34], *p* < 0.001), the N1 amplitudes decreased from the first assessment to the second assessment. The interaction between the coarse tuning effect and the EWR ability was also significant, children with lower EWR also had larger coarse tuning effect (*В* = 0.65, 95 % *CI* [0.12 – 1.18], *p* = 0.017). The main effect of trial order was also significant (*В* = −0.01, 95 % *CI* [-0.02 – −0.01], *p* < 0.001), indicating that latter trials showed stronger negativity. The interaction between EWR and Time on N1 amplitude was also significant (*β* = 0.49, 95 % *CI* [0.23, 0.75], *p* < .001), indicating that children with better EWR ability showed increased N1 amplitude at T1 but reduced N1 amplitude at T2.

Neither the main effect of age, the main effect of lexicality, nor the interactions involving lexicality were significant (all *p* > 0.05).

### 3.3 N1 lateralization

Results showed a significant right lateralization, as indicated by the intercept being significantly less than 0 (*B* = −1.48, 95 % CI [-2.33, −0.63], *p* = 0.001). This intercept represents the baseline lateralization across all conditions. None of the condition contrasts were significant (all *p* > .05), indicating no significant condition × lateralization interactions. This suggests that coarse tuning, fine tuning, and lexicality effects all showed similar bilateral distributions, with no differential lateralization patterns between conditions.

**Fig. 4:**
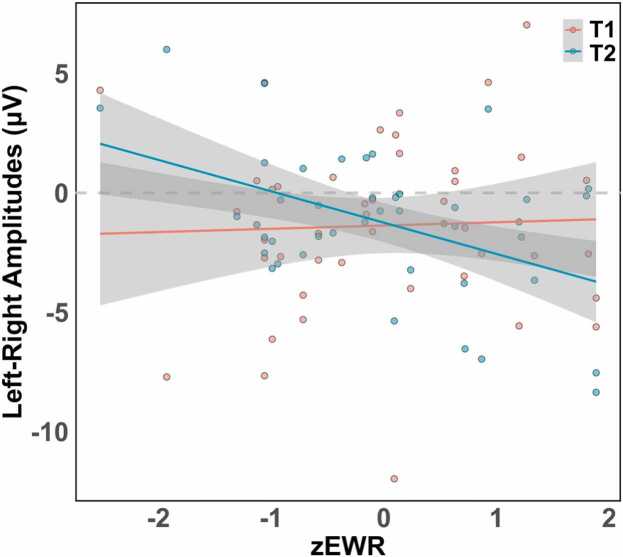
Reading ability (EWR z-scored) predicts hemispheric asymmetry in N1 amplitudes (left minus right hemisphere) at T1 and T2. Regression lines with 95 % confidence intervals shown.

There was a significant interaction between time and EWR (*В* = −1.22, 95 % *CI* [-1.62 – −0.82], *p* < 0.001), as EWR increased, children at the younger age (T1) showed more bilateral patterns, while children at the older age (T2) were more right -lateralized (see Fig. 4). The other main effects and interactions were not significant (all *p* > 0.05).

## 4 Discussion

The present study investigated the longitudinal development of N1 print tuning specialization to English as a second-learned script in native Chinese-speaking children. At the group level, our findings revealed both bilateral coarse tuning and fine tuning effects, consistent with findings from the cross-sectional study by Huo et al. (2023). The observed tuning effects to English words resemble the N1 effects identified in expert readers, indicating an emerging sensitivity to the orthographic properties of English words in second-language learners after a few years of exposure to the script (Coch and Meade, 2016, Zhao et al., 2014). Furthermore, our findings revealed that children with better English word reading abilities exhibited stronger coarse tuning at the earlier age (T1), while this trend showed a reversed direction when children were older (T2). Overall, these findings suggest that ability modulated N1 tuning for a second-learned script over time, which is further discussed below.

### 4.1 Longitudinal change of N1 coarse tuning effect

The descending developmental trajectory of coarse tuning found in this study aligns with previous EEG research in alphabetic scripts, which highlights a reduction in coarse tuning with age (Maurer et al., 2006, Maurer et al., 2011). This finding is also consistent with the previous cross-sectional study (Huo et al., 2023), which disentangled the unique contribution of age and reading abilities to coarse tuning. The current longitudinal study allowed us to investigate whether reading abilities changed over time when age was controlled, therefore enhancing our understanding of the role of reading abilities in the coarse tuning effect. This decline can be attributed to the neurobiological structural and functional changes accompanying brain development and might be indicative of the neuroanatomical correlates of typical reading development (Olulade et al., 2013, Turkeltaub et al., 2003). This would echo the neuroimaging studies demonstrating a link between cortical maturation, specifically a decrease in gray matter volume, and literacy development in children (Houston et al., 2014, Lu et al., 2007, Vandermosten et al., 2012). However, the maturation of the brain should influence the speed of both word and symbol processing to a similar extent, indicating that the observed tuning effect could reflect the increase in reading skills.

The reduction in N1 coarse tuning may signify enhanced functional efficiency within the reading-related occipito-temporal network (Price, 2010). Studies of native English speakers indicate that greater word exposure enhances the efficiency of this region (Brem et al., 2009). As children encounter more English words in both school and home environments over time, repeated practice would likely facilitate their automatic word recognition and reading fluency. Therefore, the attenuated developmental pattern of coarse tuning we observed might be related to the interplay between reading practice and concurrent brain maturation.

### 4.2 Modulation of reading ability on coarse tuning across development

We observed that English reading ability modulated the longitudinal development of the coarse tuning effect, in which the coarse tuning effect increased with EWR in the first assessment but decreased (not significantly) with EWR in the second assessment. This developmental change was observed in the L1 context (Eberhard-Moscicka et al., 2016, Maurer et al., 2011, Maurer and McCandliss, 2007), where researchers found an association between coarse tuning and reading skill for children in 1st or 2nd grade, and this association was not found when children were in 5th grade. Our findings parallel those observed in L1 contexts of the inverted U-shape during acquisition of reading skills, demonstrating that the developmental relationship between word reading ability and N1 print tuning follows a similar pattern across both L1 and L2 contexts, suggesting a common developmental trajectory in neural specialization for print regardless of language context. Our results at the first assessment were also consistent with the cross-sectional study (Huo et al., 2023), where researchers found that the coarse tuning was associated with increased reading abilities. Children in L2 contexts experience less print exposure and more limited literacy opportunities than their L1 counterparts. This is consistent with findings showing that a short period of training (Brem et al., 2018, Maurer et al., 2010, Yoncheva et al., 2010, Yoncheva et al., 2015) or repeated exposure (Brem et al., 2005) for a novel script could elicit the N1 print tuning effects. Related questions have been raised regarding whether the coarse tuning effect in L2 contexts has the same timing as in L1 contexts when readers have different degrees of exposure to visual print, or whether visually more complex print (e.g., Chinese) would delay the timing of the coarse tuning effect compared to visually simpler print (e.g., English), which requires future investigation.

On the other hand, studies of Hong Kong children reading in their native Chinese have shown that N1 print tuning is primarily driven by orthographic processing rather than phonological or semantic processing of Chinese characters (Tong, Lo, et al., 2016). These findings, combined with research on alphabetic languages, suggest that children implicitly adapt their neural processing to the specific demands of different writing systems, developing specialized responses for Chinese characters and alphabetic scripts accordingly. These findings raise the question of how Chinese children integrate two distinctly different writing systems - the alphabetic English system and the character-based Chinese system - during literacy development, and whether neural specialization that for L1 would influence L2.

### 4.3 Fine tuning and lexicality effects in L2 readers

Huo et al. (2023) previously identified a marginal age-related effect on fine tuning, but this finding was not replicated in our study. Furthermore, no evidence of developmental changes in lexicality effects was observed, adding to the mixed results documented in prior research (Coch, 2015, Coch et al., 2002, Coch et al., 2012). In the multilingual context of Hong Kong, where children are simultaneously exposed to Cantonese, Mandarin, and English scripts, the limited focus on English-specific input may impede the development of precise neural representations essential for fine distinctions in English orthography. This constrained exposure may explain the absence of fine tuning development in our findings. With regard to lexicality effects, it is possible that unfamiliar letter strings are processed as if they were novel words rather than pseudowords, reflecting a lack of automaticity in recognizing word-likeness (Smith and Dixon, 1971).

We did not observe a significant interaction between EWR and lexicality effects, diverging from earlier findings in L1 readers, as well as from Huo et al.'s results (Huo et al., 2023). This was not completely unexpected, as previous studies have reported the absence of lexicality effects for non-native children and adult learners (Eberhard-Moscicka et al., 2016, Tong et al., 2016, Wang et al., 2023). Moreover, this non-interaction remained consistent over time, showing no developmental changes in their association, aligning with the cross-sectional findings of Huo et al. (2023). This may suggest that the relationship between reading ability and lexicality effects may be less robust for L2 readers, possibly indicating that proficient L2 children remain less attuned to such distinctions regardless of ability. Interestingly, the lack of a relationship and developmental changes between EWR and fine tuning that we found is consistent with the findings of (Huo et al., 2023). One plausible explanation is that fine tuning is specific to processing well-formed letter patterns, and the word decoding demands of the EWR task, similar to Huo et al.'s study, may not directly tap into this process. In contrast, coarse tuning might play a broader role in supporting word decoding, thus explaining its stronger association with EWR.

Altogether, our results do not completely support findings from L1 studies which demonstrated the association of word reading abilities with increased N1 tuning to English words (Brem et al., 2018, Coch and Meade, 2016, Maurer et al., 2011). Instead, our findings highlight how word reading ability contributes differently to coarse tuning, fine tuning, and lexicality effects in L2 readers. These findings suggest that L2 readers may experience a delay in the emergence of later-stage N1 sensitivity, highlighting the challenges posed by limited exposure and the differential development of fine tuning and lexicality effects in L2 English readers.

### 4.4 Lateralization of the coarse tuning effects

Previous research (Huo et al., 2023, Tong et al., 2016) demonstrated right-lateralized N1 coarse tuning effects, with younger skilled readers showing stronger right lateralization for symbols versus real words—interpreted as suppression of non-print stimuli. Our longitudinal study yielded different results: while we observed overall right-lateralized N1 distribution modulated by time and reading ability, we found no condition-specific lateralization effects. The bilateral distribution across all print conditions and the non-significant interaction between condition and hemisphere indicate that we did not replicate the symbol suppression effect at either timepoint.

Notably, our sample represents a longitudinal subset of participants from the cross-sectional study by Huo et al. (2023), yet we observed divergent topographic patterns for coarse tuning effects despite examining the same L2 learner population. This discrepancy suggests that the condition-specific lateralization observed in cross-sectional comparisons may not be stable within individual developmental trajectories. In longitudinal designs, age and reading ability may exert opposing influences on lateralization within the same developing reader: while increasing age promotes more bilateral neural organization (Maurer et al., 2011), advancing reading skills may promote greater specialization. These counteracting forces could cancel out condition-specific lateralization effects at the group level. Alternatively, the non-print suppression effect observed in cross-sectional L2 samples may reflect individual differences that are present at specific developmental snapshots but do not represent consistent within-child developmental changes. Together, these findings suggest that condition-specific coarse tuning lateralization in L2 contexts may be more variable and context-dependent than previously assumed.

## 5 Conclusion

The present longitudinal study revealed that coarse tuning changes over time, and this tuning effect was modulated by reading ability over time in an L2 context. It showed similarities with the cross-sectional study, with a longitudinal decrease in coarse tuning effects. However, it also differed in that it showed that the longitudinal change was modulated by reading skills. In conclusion, our study highlights the dynamic nature of neural print tuning in second language learning, demonstrating that the coarse tuning effects are particularly sensitive to reading development.

## CRediT authorship contribution statement

**Shuting Huo:** Writing – review & editing. **Jason Chor Ming Lo:** Software, Methodology, Investigation, Conceptualization. **Catherine McBride:** Writing – review & editing, Resources, Funding acquisition. **Urs Maurer:** Writing – review & editing, Supervision, Resources, Funding acquisition. **Xin Huang:** Writing – original draft, Visualization, Formal analysis. **Jai Dellosa Ariza:** Writing – original draft, Visualization.

## Declaration of Competing Interest

The authors declare the following financial interests/personal relationships which may be considered as potential competing interests: Urs Maurer reports financial support, article publishing charges, and equipment, drugs, or supplies were provided by University Grants Committee Research Grants Council. Catherine McBride reports financial support was provided by University Grants Committee Research Grants Council. If there are other authors, they declare that they have no known competing financial interests or personal relationships that could have appeared to influence the work reported in this paper.

## Data Availability

Data will be made available on request.
