# Supplementary material for: Neural specialization of print processing in second language learning: A longitudinal ERP study of Chinese children learning English
Source: Dev Cogn Neurosci. 2026 Feb 10;79:101691. doi: 10.1016/j.dcn.2026.101691 (PMC12925297; doi:10.1016/j.dcn.2026.101691)
Supplement: Supplementary file 1 — Supplementary material [file mmc1.docx]

Table 1. *Fixed Effect Estimates from Linear Mixed-Effects Model of N1 Amplitude Including Home English Use as a Covariate*

| Factor | N1 amplitude | | | |  |  |
| --- | --- | --- | --- | --- | --- | --- |
|  | *b* | *CI* |  | Sign. |  |  |
| (Intercept) | -3.69 | -6.13 – -1.25 |  | **0.003**** |  |  |
| zAge | -0.79 | -1.88 – 0.30 |  | 0.154 |  |  |
| HomeEnglish | -0.04 | -1.34 – 1.26 |  | 0.951 |  |  |
| zOrder | -0.01 | -0.02 – -0.01 |  | **<0.001***** |  |  |
| Time | 2.02 | 1.75 – 2.29 |  | **<0.001***** |  |  |
| Coarse-tuning | 4.46 | 3.78 – 5.13 |  | **<0.001***** |  |  |
| Lexicality | 0.03 | -0.65 – 0.70 |  | 0.940 |  |  |
| Fine-tuning | 1.12 | 0.45 – 1.79 |  | **0.001***** |  |  |
| zEWR | 0.07 | -0.95 – 1.08 |  | 0.894 |  |  |
| Time × Coarse-tuning | -1.26 | -2.01 – -0.50 |  | **0.001***** |  |  |
| Time × Lexicality | 0.30 | -0.45 – 1.05 |  | 0.432 |  |  |
| Time × Fine-tuning | -0.22 | -0.97 – 0.53 |  | 0.563 |  |  |
| Time × zEWR | 0.43 | 0.16 – 0.70 |  | **0.002**** |  |  |
| Coarse-tuning × zEWR | 0.49 | -0.07 – 1.05 |  | 0.084 |  |  |
| Lexicality × zEWR | -0.12 | -0.68 – 0.43 |  | 0.660 |  |  |
| Fine-tuning × zEWR | -0.32 | -0.88 – 0.23 |  | 0.258 |  |  |
| Time × Coarse-tuning × zEWR | -0.84 | -1.60 – -0.08 |  | **0.030*** |  |  |
| Time × Lexicality × zEWR | -0.28 | -1.03 – 0.48 |  | 0.471 |  |  |
| Time × Fine-tuning × zEWR | 0.24 | -0.51 – 1.00 |  | 0.527 |  |  |

* *p* < .05. ** *p* < .01. *** *p* < .001.

Table 2. *Fixed Effect Estimates from Linear Mixed-Effects Models Examining Lexicality Effects on N1 Amplitude*

| Factor | N1 amplitude | | | |  |  |
| --- | --- | --- | --- | --- | --- | --- |
|  | *b* | *CI* |  | Sign. |  |  |
| (Intercept) | -4.04 | -5.36 – -2.72 |  | **<0.001***** |  |  |
| zAge | -0.80 | -1.93 – 0.33 |  | 0.164 |  |  |
| zOrder | -0.01 | -0.02 – -0.01 |  | **<0.001***** |  |  |
| Time | 2.11 | 1.85 – 2.37 |  | **<0.001***** |  |  |
| Condition | 0.06 | -0.84 – 0.97 |  | 0.890 |  |  |
| zEWR | -1.63 | -3.19 – -0.07 |  | **0.040*** |  |  |
| zEVK | 1.69 | 0.11 – 3.28 |  | **0.036*** |  |  |
| Time × Condition | 0.36 | -0.37 – 1.10 |  | 0.332 |  |  |
| Time × zEWR | 1.82 | 1.24 – 2.41 |  | **<0.001***** |  |  |
| Condition × zEWR | 0.68 | -0.52 – 1.87 |  | 0.268 |  |  |
| Time × zEVK | -1.46 | -2.04 – -0.88 |  | **<0.001***** |  |  |
| Time × Condition× zEWR | -0.78 | -2.43 – 0.86 |  | 0.352 |  |  |
| Time × Condition× zEVK | 0.70 | -0.93 – 2.33 |  | 0.400 |  |  |

* *p* < .05. ** *p* < .01. *** *p* < .001.

Note. Condition represents the comparison between real words and pseudowords (lexicality effect).

## **Behavioural Measures**

English rapid digit naming: This rapid automatized naming task, adapted from Denckla and Rudel (1976), assessed children's naming speed in English. The task consisted of a chart with eight rows of five randomly ordered digits (2, 4, 6, 7, and 9), with each digit appearing multiple times across the array. Children were instructed to name all digits aloud in English as quickly and accurately as possible. Two trials were administered, and the average completion time across trials (in seconds) was used as the performance measure. The inter-trial correlation was 0.86, indicating high reliability.

English vocabulary knowledge: A task similar to the one used for assessing children's Chinese vocabulary knowledge (e.g., Tong et al., 2018) was administered to measure children's English vocabulary knowledge. The task included three parts: English receptive vocabulary, expressive vocabulary, and vocabulary definitions. All test items were in English. There were 21, 23, and 26 test items for the receptive, expressive, and vocabulary definition parts, respectively. Children stopped once they gave five consecutive incorrect responses. The English vocabulary definitions part comprised 15 vocabulary items arranged in order of increasing conceptual difficulty. Each word was presented orally, and children were asked to explain its meaning. A 0- to 2-point standardized marking scheme was used to score responses. No answer or an obviously incorrect answer was scored as 0, while a comprehensive description of the target word was scored as 2. A score of 0.5 was given for a correct Chinese translation of the English word. The rationale for this coding scheme was that if children could translate an English word into Chinese, it reflected their ability to understand the word, but they were not sufficiently proficient in English to explain it. The strongest skill would be to both understand and explain the word in English. The maximum possible score for this part was 30. The same stopping rule (five consecutive incorrect answers) applied. The internal consistency reliability for this test was .94.

English dictation: English spelling ability was measured through a dictation task requiring children to write 12 compound words. To parallel the two-character Chinese dictation task, compound words were deliberately chosen as test items. Scoring allocated one point for each correctly spelled component word within the compound, yielding a maximum possible score of two points per item and 24 points in total.
